# Adolescent Life Perspectives After War: Evaluation and Adaptation of the Future Expectation Scale in Uganda

**DOI:** 10.3389/fpsyg.2019.01527

**Published:** 2019-07-03

**Authors:** Laura B. Saupe, Katharina Gößmann, Claudia Catani, Frank Neuner

**Affiliations:** Clinical Psychology and Psychotherapy, Department of Psychology, Bielefeld University, Bielefeld, Germany

**Keywords:** future expectations, adolescence, Uganda, post-conflict, factor structure

## Abstract

The formulation of life perspectives is one of the developmental tasks of adolescence. Expectations regarding one’s own future are shaped by cultural and contextual factors. However, there is little cross-cultural research that includes countries affected by war and turmoil. A Ugandan version of the Future Expectations Scale for Adolescents (FESA) was developed and evaluated with a sample of 279 Ugandan adolescents with low socioeconomic status living in rural communities affected by the Ugandan civil war (1986–2006). The Ugandan FESA was constructed on the basis of a combined item pool of the original Chilean and an adapted Brazilian FESA. Confirmatory factor analysis revealed that the factor structure of the original FESA did not fit the Ugandan data. Principal component analysis revealed a 3-factor solution, including the domains of children and family, work and education, and general future optimism. The final version consists of 19 items, which were deemed culturally appropriate by local focus groups. Overall, the item pool of the FESA was found useful for further studies in post-conflict societies.

## Introduction

Worldwide, adolescents’ views on their impending transition to adulthood vary. These perceptions and expectations are covered in part by the concept of future orientation, which constitutes the aspect of juvenile identity that is related to the development of future-oriented interests as well as to the commitment to goals associated with future outcomes (Nurmi, 1991). According to Nurmi (1991), future orientation consists of three facets: motivation (future interests), planning (realization of future interests), and evaluation or expectations (beliefs around whether and to what extent these future interests will be realized). The third component relates to the individual’s expectations about the extent to which major developmental tasks such as school completion, marriage, parenthood, and the beginning of one’s career will be successfully mastered. The study of future expectations is relevant as it has been associated with future outcomes; for example, holding low future expectations about one’s ability to achieve major goals in life is associated with widespread risk-behaviors (Harris et al., 2002; Valadez-Meltzer et al., 2005), including early parenthood (Thompson et al., 2012; Thompson and Neilson, 2014).

While the general aims of future expectations – such as reaching certain family planning or career goals – have been remarkably consistent across cultures, the relative importance of specific topics and one’s optimism about achieving these different developmental milestones differ greatly between samples varying in culture, gender, and age (Nurmi, 1991; Shanahan, 2000; Albert and Trommsdorf, 2014; Peou and Zinn, 2015). In addition to social influences that are more or less consistent within cohorts, family characteristics have an impact on future expectations in adolescents. For example, psychological stress caused by child maltreatment, adverse parenting styles, a negative family climate or low socioeconomic status (Nurmi, 1991; Thompson and Neilson, 2014) tends to leave those exposed with lower levels of conviction that they will complete major developmental tasks.

Across birth cohorts, trends concerning the fulfillment of the typical developmental tasks are influenced by short-term economic factors and discrete historical events such as wars or political transformations of societies. Within the same birth cohorts, social inequalities relating to gender, race, and socioeconomic status have been found to play an influential role in the course of development. As a consequence, it is probable that an unfavorable combination of inter- and intra-cohort factors defines subgroups that are especially at risk for negative long-term development, including a more negative view of their future prospects (Shanahan, 2000; Peou and Zinn, 2015).

Over the past half century, the global expansion of access to formal education, women’s increased participation in the labor market, and a decline in job security have led to individualization of the order and the age by which adolescents reach the different markers of adulthood. This includes milestones such as getting married, becoming a parent and entering the job market (Arnett, 2000). The individualization of the adulting process of reaching adulthood has increased a sense of uncertainty, particularly for adolescents in societies transitioning in the wake of war and conflict (Peou and Zinn, 2015). This uncertainty leaves youths living in these societies torn between hopes for a better life in times of peace, the potential of greater economic growth, and a new-found political stability while still dealing with the daily stressors of post-war life such as poverty, lost infrastructure, heightened levels of violence, and decreased physical and mental health (Bozzoli, 2010; Bozzoli et al., 2010a,b, 2011; Peou and Zinn, 2015). Nevertheless, little is known about how mass-violence such as war and armed conflicts, as well as the economic and social turmoil which follow in the wake of a civil war, impact youths’ future expectations. This lack of investigation is notable, as these individual expectations may play a vital role in the reconstruction of a nation (Baines et al., 2006). Therefore, research should take the individual future expectations of young adults in post-conflict societies more strongly into account. However, there is a lack of tools to measure future expectations in such a context.

An outstanding example of a post-conflict region in Africa is Northern Uganda, where the present study took place. The country has undergone massive changes after years of violent conflict. It has been struck by a civil war between the Lord’s Resistance Army (LRA) and the Ugandan military from 1986 to 2006. The civilian population was fell victim to numerous raids and abductions by the LRA, which led to their forced internment in camps for internally displaced people (IDP) from 1996 onward. In 2003, nearly 90% of the civil population in the Acholi region lived in IDP camps (International Crisis Group, 2006; Bjorkhaug et al., 2007). Levels of posttraumatic stress, depression, and alcohol-related symptoms were high within this IDP population (Roberts et al., 2008, 2011; Ertl et al., 2014). Life in the camps was also heavily influenced by the erosion of familial and social relationships (Hovil and Moorhead, 2002), high levels of intimate partner violence (Uganda Bureau of Statistics, and Macro International, 2007), and the fact that 27% of all children had lost at least one parent due to the war (Uganda Bureau of Statistics, and Macro International, 2007).

Since 2006, people in the Acholi region have been slowly returning to their home villages, trying to restructure village and family life while still dealing with the massive changes and stressors of the war and post-war years. Research from countries with similar conflicts shows that the post-war years are often marked by land disputes leading to violence and social disorder within communities (Betancourt et al., 2014), economic hardship (Klasen et al., 2010), as well as a rise in family violence (Catani et al., 2008; Panter-Brick et al., 2009; Klasen et al., 2010). Results of a larger research project that includes the present study’s sample (Saile et al., 2013a,b, 2015) demonstrated that family life was heavily influenced by high levels of intimate partner violence, child abuse, and hazardous drinking in men. In addition, children and their parents reported high exposure to a multitude of traumatic events, both during and after the war. These findings illustrate that northern Uganda is currently coping with the typical stressors and uncertainties of a post-conflict society, making it an exemplary setting to evaluate adolescents’ future expectations in the context of a transforming society. Currently, 69% of the Ugandan population are under the age of 25 (Central Intelligence Agency, 2018). As these children and adolescents will play a vital role in rebuilding and restructuring their villages and influence the future of their country, measuring their hopes and fears for their own future seems all the more important.

One of the pioneering studies of northern Ugandans’ future expectations was conducted by Bozzoli et al. (2010a). The authors showed the negative influence of conflict on individual expectations concerning future economic circumstances and, to some extent, on individual welfare. However, this study was limited since future expectations were assessed with a single item, rating the expectations as to what extent the participants’ economic future/general lives would improve, deteriorate, or stagnate over the course of the next year. This study did not provide a more nuanced insight into different domains of future expectations.

Taking the current literature on adolescent future expectations into account, more specific aspects of future expectations should be studied in detail, since cultural and societal factors play a critical role in determining the importance of different aspects of life (such as family planning, religious life, health, education, and occupation; Nurmi, 1991, i.a.). More elaborated instruments, such as the *Future Time Perspective Scale* (Carstensen, 1996) and the *Future Orientation for Adolescents Questionnaire* (Seginer, 2000, 2008) define future expectations as a function of anticipated achievements divided into the two areas of *family* and *career*. This distinction falls short of considering further areas of future expectations (such as religion, health, or community life; see Nurmi, 1991) that have been proven to be relevant across cultural backgrounds. Since it is unclear to what extent the expectations in these various domains are intercorrelated and sensitive to large-scale social events, a more sophisticated instrument that assesses different areas separately and also works in non-Western societies is needed.

The Future Expectation Scale for Adolescents (*FESA*) developed by McWhirter and McWhirter (2008) as a multifaceted instrument fulfills this criterion: The *FESA* is a 24-item questionnaire measuring children’s and adolescents’ expectations for their future in the domains of *work and education*, *children’s future*, *marriage and family*, *health*, and *church and community*. It was originally applied in a Chilean sample of adolescents living in a poor, working-class neighborhood. The authors were interested in developing the *FESA* in a Chilean sample since Chile had undergone a massive transformation process after the 1973 military coup and subsequent 17-year dictatorship. In their evaluation sample, they found an internal consistency of α = 0.87 for the complete scale, with a range from 0.71 (*health*) to 0.88 (*work and education*). The five-factor solution explained 59.9% of variance. In their evaluation sample, the *FESA* sum-score correlated positively with hope, educational variables, parental monitoring and connectedness to the self, others, and society. The authors found negative correlations of the sum-score with perceived barriers of education. Differential findings were reported for the *health* subscale, which was negatively correlated with risk factors such as alcohol consumption and smoking. The *FESA* was then adapted for use in a sample of Brazilian teenagers and young adults of different socioeconomic statuses (Dutra-Thomé et al., 2015). However, as the original *FESA* factor structure did not fit the Brazilian sample, the authors changed some of the items and calculated a different factor structure. Some items loaded better onto other factors, whereas others were deleted as they did not fit the Brazilian sample. The authors also added items concerning *church and community*. The final Brazilian version of the *FESA* consisted of 22 items and a 5-factor solution, which explained 59% of variance. For a detailed description of the changed item pool and factor structure, refer to Table 2.

It thus seems that while the *FESA* is an adequate measure to explore adolescent future expectations in emerging countries, cultural and societal factors have an influence on the questionnaire’s items and factor solution. Both author teams emphasize in their discussion the necessity of utilizing the *FESA* in a variety of cultural and societal contexts to evaluate the need for adaptation. Neither Chile nor Brazil has been involved in an armed conflict during the years immediately prior to the *FESA* evaluations studies. They differ therefore in their economic development, social structure, and level of infrastructure from countries that are only just recovering from war and displacement, such as Northern Uganda. As living in post-conflict societies seems to influence the concept of future expectations (Baines et al., 2006; Bozzoli et al., 2010b, 2011), it is fair to assume that the *FESA* structure and item pool would need to be adapted in such a setting.

The aim of the present study is therefore, to evaluate whether the *FESA* can be applied in the post-conflict setting of Northern Uganda, and how this setting influences the questionnaires’ item and factor structure. We expect that the factor structure of the original Chilean *FESA* will not be applicable in the Ugandan setting, but that the individual items can be applied.

## Materials and Methods

### Setting and Participants

The sample of the present study is part of an ongoing research project focusing on family life, childhood and early adulthood in a post-war society. The project consists of three waves of interviews (2010, 2012, and 2016) with Ugandan students living in seven rural communities who were affected by the war between the Ugandan government and the LRA (Saile et al., 2013a,b, 2015). Of the 360 s-grade students who were interviewed in 2010, 285 (158 males and 127 females) now adolescent students were re-contacted and agreed to participate in the 2016 wave of data collection, which included the FESA evaluation study. This equals a response rate of 79.2% of the original sample interviewed in 2010, which is comparable to retrieval rates after 6 years in similar research samples (Betancourt et al., 2013; Panter-Brick et al., 2014).

Dropouts between 2010 and 2016 were mainly cases in which students could not be located again (*n* = 57, 15.8%), had moved too far away to be interviewed again (*n* = 8, 2.2%), had passed away (*n* = 4, 1.1%) or were physically too sick to be interviewed (*n* = 6, 2.2%). There were no significant differences between those adolescents who participated in 2016 and those who dropped out. All adolescents who were reached in 2016 agreed to participate in the present study.

### Translation and Data Collection Process

All instruments were translated into the local language of Luo Acholi according to recommended procedures in transcultural research (Flaherty et al., 1988; van Ommeren et al., 1999) including translation, lexical back-translation, blind back-translation, and separate focus group discussions with bilingual local therapists. The questionnaire used for the present analysis included a sociodemographic section that captured individual and household characteristics and the Ugandan *FESA* as described in the next section. All questions were administered in a one-on-one interview format in the local language Luo-Acholi by trained local therapists under the supervision of an international team of clinical experts with extensive experience in transcultural research, each of whom had obtained a master’s degree or higher in clinical psychology. The 14 local therapists (5 male and 9 female) had on average 7.14 years (range 2–11 years) of clinical, diagnostic and research related experience. They were all trained for 4 weeks in the administration of the interview by the international team of clinical experts.

To avoid effects possibly caused by altering questionnaire administration, all questionnaires were administered as interviews to take the differences in the literacy of the participants into account. Accordingly, the chosen language for all interviews was Luo Acholi as the proficiency of the English language varied widely between the participants.

Interviews were conducted on the school grounds, in either empty classrooms or in secluded areas under a tree on the school compound with only the interviewer and the student present. Interviewers read the instruction for the *FESA* in Luo Acholi aloud (translated: “Now, I will read some statements concerning your future to you. Please decide how true each statement is for you in your situation with 0 meaning “I do not believe this at all” and 4 meaning “I certainly believe this”). For each question, interviewers read the verbalization of the different scale points of the *FESA* before asking the student to indicate their answer by either answering verbally or pointing on a graphical representation of the scale.

### Focus Groups and Cultural Adaptation

As a first step of the adaptation process the key instruments and theoretical concepts such as adolescence, future expectations and future planning, and changes in community support after the war in Acholi culture were discussed in three different focus groups. One focus group consisted of twelve local clinical counselors, the second one of eight teachers of rural schools and the third one of ten urban teachers. All focus group discussions were led by an expert in clinical psychology and conducted in the local language Luo Acholi which was interpreted into English by an experienced translator. The results of the focus group discussions revealed important information for the adaptation of the instrument to ensure validity in the context and were interpreted in the context of current research as follows.

*Adolescence* was initially defined by the focus groups as the timeSpan between childhood and adulthood, approximately between 13 and 17 years of age, when young people start to explore their opportunities and “are trained to take over more responsibilities within their families or communities,” often under the leadership of a respected adult. The end of adolescence was defined as the point were “they go and live with a partner in their own hut,” as this would mark the start of a new family within the community. None of the focus groups mentioned any initiation rituals for girls or boys to mark the transitioning from childhood to adolescence, which is in accordance with other studies in Northern Ugandan Acholi communities (Lundgren, 2014; Vorhölter, 2014). It thus seems as if marriage is commonly seen as the end of adolescence. Currently, the mean age in Uganda to get married for girls is 20 years and 24.3 years for boys (WorldBank, 2011). Lately, the trend to frown upon young marriages and to emphasize the importance of “education first – marriage later” as part of the school and church curriculum has also reached rural areas. All students who were interviewed in our study said they had discussed the subject of later marriage as the more respectable option with their teachers or elders. The focus groups revealed the predominant view that the length of childhood and adolescence in this setting was highly dependent on the parents’ financial and emotional ability to provide their offspring with a time period where they are not already required to fully take over adult responsibilities. In rural communities, girls were considered as adults slightly earlier than boys. A review on the meaning of adolescence in 187 societies spread all over the globe comes to the conclusion that a universal stage of adolescence as the time between childhood and adulthood exists, marked by distinct characteristics that differ from the preceding and following developmental stages in the way adolescents behave and are treated (Schlegel and Barry, 1991). It seems that the meaning of adolescence in Uganda meets the global criteria for this developmental phase, as an age of identity exploration, instability, self-focus and the exploration of possibilities (Arnett, 2014). This is in accordance with the review of Raffaelli et al. (2013) who claim that the Western concept of adolescence is mainly applicable in Africa – but further research should look into the individual differences in conceptualization of this developmental period in regards to living conditions in the different African nations.

Granting these cross-cultural aspects of adolescence, certain specificities due to the societal influences have to be noted (Schlegel and Barry, 1991; Dasen, 2000): young people from individualistic societies seek to enhance their own agency to become more independent, whereas youth in sub-Saharan Africa who grow up in societies with a more collectivistic culture seek to become part of a larger collective (Cole, 2011). Assessing future expectations of adolescents in a collectivistic society should thus include the domains of community and family life. It has also been noted, that up to now the influence of globalization, increased access to formal education and the entry into a more commercial market have changed the interpretation of adolescence in former traditional societies to more individualistic socialization (Raffaelli et al., 2013). Comparably to the West, adolescents in Uganda and elders are seen as very distinctive categories with different characteristics and – most importantly – different agencies: adolescence is the time were young people start to evaluate possible career paths and positions within the community they want to fulfill, but still have to refer to senior members of their kin (Vorhölter, 2014). It thus seems that the meaning of adolescences in Northern Uganda is part of the ongoing change of Acholi culture due to the war, displacements, and resettlements, as well as the changing influence of a former collectivist culture to a more individualistic approach to growing up (Caldwell et al., 2006). This paper is built on the assumption that adolescence as a global concept of the period of time where young people explore their future possibilities and find their voice within or outside their communities is applicable in the Ugandan context, as long as the emphasis on family values and community involvement are considered, which is in correspondence with the definition of adolescence in the Ugandan National Youth policy as “… a period of great emotional, physical and psychological change that requires societal support for a safe passage” (Republic of Uganda, 2001).

Secondly, the focus groups agreed that future expectations of young people in their communities in the form of hopes and dreams for improving life for oneself and the next generation are considered to be the best way “to show you are serious about life.” Nevertheless, many mentioned that issues related to *future expectations/future planning* were rarely discussed within the school context or between parents and children. Especially in rural areas, day-to-day survival was said to leave little time, energy, or financial resources for families to discuss such matters with their children. Among adolescents, these subjects are most likely to be talked about at the end of 6th grade, which marks the end of primary school since the biggest obstacle to any future plans beyond farming typically centers on getting access to secondary school. Religious beliefs were said to be closely related to future expectations, leading to the general conviction that in the end, it would all be up to a higher power (“God”), but hoping for a better future was a first step to improving one’s life. These results of the focus groups are in tune with the ongoing discussion whether the Western concept of the linearity of time can be compared with the circular time perspective in most African countries. Lately, this dichotomy has been criticized as simplifying the understanding of time and future planning in Africa: following Munn (2002), the so-called circular or repetitive time (as in the recurrence of the seasons) does not necessarily preclude a linear understanding of the passage of time. She argues that people are in a sociocultural time of multiple dimensions, including circular events (as in seasons) as well as past-present relations. Although culture influences the sense of temporality, Uganda has been caught between the traditionalist society and Western influence for centuries, making the linear time perspective more common than often assumed (Adjaye, 2002). Adolescence and future expectations, also referred to as *lived time*, are experienced as the linear and irreversible time in which an individual experiences the passage from childhood to adulthood despite cultural influences (Saulawa, 1989). Crane (2013) goes even further: she cautions that the dominant Euro-American view that concepts like time and future planning in African nations are too profoundly different to the Western concepts to be compared, have led to serious consequences independent of any relationship to empirical evidence. One example would be the withholding of anti-retroviral treatments for HIV positive Africans, because of the Western view that the lack of a linear time perspective and future planning would prohibit African patients to take their medication as prescribed. This happened despite later studies providing empirical evidence, that the adherence rate in Uganda and other African nations (90%) were much better than in the United States (70%). However, it can’t be denied that cultural and historical circumstances influence the perception and meaning of time and future. According to a study in Eastern Uganda, the historical linear passage of time is often reframed in rural areas as time related to other people, linking the ancestors to future children (Whyte and Whyte, 2004). While it seems reasonable to assume that the concept of future expectations as a linear concept of time is applicable in a rural Ugandan setting, any instrument measuring future expectations should take this intersubjectivity into account by asking about future family plans and relations to family members. The latter is one of the strengths of the *FESA*. Another aspect to be considered is, that although future planning plays a role in the lives of young people in rural Uganda, the decision making process of all people consists of an individual weighting of a past (past experiences), present (what is happening right now) or more future (expectations and hope) oriented appraisal process (Zimbardo and Boyd, 1999). Especially hard living conditions seem to be linked to bias toward stronger present orientation, although this does not negate the existence of future planning (Jones and Brown, 2005). According to our focus group discussions, it might well be that the appraisal of the present situation bears a stronger weight than complex reflections on future outcomes in the rural post-war context of the present study’s sample. This could result in less detailed future expectations compared to the Chilean and Brazilian *FESA*.

We therefore take the stance that exploring future expectations in a Northern Ugandan sample can be done on the basis of the existing *FESA* item pool while keeping in mind that the influence of the social aspect of experience of time and the individual weighting process in the appraisal of time might influence the structure of the instrument in Uganda.

Third, the biggest *change in community support and structure* since the war was described as a decrease in shared communal life and responsibilities. Before the war communities were described as fairly close-knit, with elders being in charge of all children and adolescents in their proximity. They provided guidance and social control over the younger generation regardless of kinships. Field and community work were shared among all members of the community, increasing the sense of interdependence. Since the war and the displacement of most villagers in Northern Uganda, most families fend for themselves, leaving the supervision and guidance of offspring to the individual nuclear families. Clan members exercise their monitoring roles less routinely, but might still use their authority in cases of adolescent behavior that severely disrupts family or communal life.

### Ethics Statement

The ethics committee of the German Research Foundation (DFG), the ethics committee of Gulu University in Uganda, and the National Council for Science and Technology in Uganda (UNCST) approved of the study protocol. All participants and their primary caregivers were invited to an information meeting where the study’s objectives and procedures were explained. Written consent of at least one primary guardian and the participating adolescent were collected. No monetary incentives were given, but all participants received a snack during the interview. After completion of the study, all children who had participated in the study in 2010 and their guardians were invited to take part in a community training that involved fostering improved parenting skills (female guardians) and dealing with domestic violence and suicidal ideation (adolescents), independent of their participation in the data collection at the second (2012) or third (2016) wave.

### Development of the Ugandan Version of the FESA

The Ugandan version of the *FESA* was developed by listing all *FESA* items of the Chilean and the Brazilian version, resulting in a total item pool of 27 items (see Table 2). Each item was then assessed for their adequacy in the northern Ugandan rural setting in a discussion between two clinical experts with extensive experience in transcultural research in a Ugandan setting and a group of local counselors with extensive experience with youths and young adults in the communities. This led to the deletion of five items. Two of the items were deleted because they were deemed unfit for a rural Ugandan population (“I will participate in sports”; “I will do voluntary work in my community”). In addition, two items concerning religion and faith (“I will participate in many church activities,” “I will instill faith in my children”) were omitted because local counselors argued that religious life in rural areas of northern Uganda is neither as organized as the word “activities” implies nor is it seen as a matter of choice whether to raise children with a sense of Christian faith. The item “I will get married before the age of 25” was deleted because marriage before the age of 25 is so common in rural areas that the authors expected a lack of variance in participants’ answers. The final item-pool of the Ugandan version of the *FESA* prior to the analysis consisted of 22 items (see Table 2). 21 of the items were also part of the original Chilean *FESA* and one item was only used in the Brazilian *FESA*. The answer format of the 5-point Likert scale ranging from “I do not believe this at all” to “I certainly believe this” of the Brazilian *FESA* and the revised version of the original Chilean version of the *FESA* (see Dutra-Thomé et al., 2015 for the psychometric details of the revised version of the original Chilean *FESA*) was applied. The 5-point-Likert scale was chosen instead of the original Chilean 7-point Likert Scale, as the local research team argued that participants in the rural areas tend to struggle with more detailed scales.

### Statistical Analysis

Means, standard deviations, ranges, and frequencies were calculated on the basis of the unweighted data from the whole sample to describe the sample characteristics.

Confirmatory factor analysis (CFA) was performed to evaluate whether the structure of the original Chilean *FESA* (McWhirter and McWhirter, 2008) fit the Northern Ugandan data using SPSS AMOS 25. The Model fit of the analysis was tested according to the criteria for model fit indices suggested by Schermelleh-Engel and Müller (2003) and Schweizer (2010). As the Chi-square test has been criticized as being too sensitive to large sample sizes (Bonett and Bentler, 1980), additional fit indices including Comparative Fit Index (CFI), Tucker-Lewis Index (TLI), Incremental Fit Index (IFI), Root-Mean-Square Error of Approximation (RMSEA), and Standardized Root-Mean-Square Residual (SRMR) were used. Model fit was considered to be acceptable if the calculated model featured TLI, IFI, and CFI values of ≥ 0.90, an RMSEA value of ≤ 0.06 with 90% confidence interval values < 0.05 (lower value) and < 0.08 (upper value), and an SRMR value below 0.08. In addition, factor loadings should be well above 0.50. As the focus group had deemed the items “I will participate in many church activities,” “I will get married before I am 25 years old” and “I will participate in sports or another type of exercise” of the original Chilean *FESA* sample inappropriate for the Ugandan context, these items were not included in the questionnaires and could thus not be assessed in the CFA. Additionally, the item “I will dedicate time to spend with my family” used in the Ugandan version of the *FESA* that was only part of the Brazilian (Dutra-Thomé et al., 2015) but not the Chilean version (McWhirter and McWhirter, 2008) of the *FESA*, was excluded from CFA. Since the item-pool of the Ugandan version differed only slightly from the Chilean *FESA* (3 items differed) but more strongly form the Brazilian item pool (6 items of the Ugandan *FESA* were not included in the Brazilian version) we did not calculate the model fit for the Brazilian *FESA* structure in the Ugandan sample.

Since we did not expect an acceptable model fit of the original Chilean factor structure for the Ugandan data, the *FESA* factor structure of the Ugandan sample was further examined with principal component analysis (PCA). PCA was chosen as an adequate (Kline, 1994; Field, 2011) and the most commonly used (Guadagnoli and Velicer, 1988; Watson and Thompson, 2006) statistical method to reduce a larger number of items to fewer underlying dimensions. In a few cases, PCA has even been found superior to exploratory factor analysis in eliminating redundancy in the development of questionnaire scales (Krishnan, 2011). All 22 items of the *FESA* were entered simultaneously as independent variables in the first round of the PCA. As intercorrelations between the single main components were expected and were consistent with the theoretical model, oblique rotation (direct oblimin) was chosen. Sample adequacy was calculated with the Kaiser-Meyer-Olkin measure (KMO). In addition, Bartlett’s test of sphericity was used to prove that correlations between items were sufficiently large for PCA. Only components with eigenvalues of Kaiser’s criterion > 1 were included. After a first examination of the results of the PCA with all items, the component structure was discussed between transcultural experts on theoretical grounds. After the discussion, items that did not match the Ugandan context were excluded and the PCA was run again. The resulting component structure was used to calculate the sample’s mean, standard deviation, and range, as well as the internal consistency (Cronbach’s α) of the whole resulting *FESA* scale as well as of each component. Intercorrelations between main components were calculated using Spearman’s *rho*, one-tailed.

The statistical analyses concerning the PCA as well as the descriptive analyses were conducted using SPSS version 24.

## Results

### Demographics

The average age of participants in our sample was *M* = 14.59 years (*SD* = 1.41, range 11–19 years). Females were slightly younger (*M* = 14.33 years, *SD* = 1.42) than males (*M* = 14.8 years, *SD* = 1.37). The majority of the students (*n* = 242, 84.9%) were still in school. Most of them attended the 5th grade of primary school (44.6%) and had on average proceeded through 2.25 grades over the past 6 years. For a more detailed description of class attendance in 2016 refer to Table 1. Most students had to at least temporarily drop out of school because their parents needed them to do fieldwork or household chores (*n* = 114, 40.1%) or due to lack of money for school fees (*n* = 69, 24.3%). Nearly half of the students (*n* = 135, 47.4%) lived with both of their biological parents, whereas 30 (10.5%) lived only with their father, 59 (20.7%) lived only with their mother, and 60 (21.1%) lived with people other than their biological parents. One (0.3%) student lived by himself.

**TABLE 1:** Current grade of students participating in the 2016 study.

	**Current grade**	***n* (%)**
	4	32 (11.2)
	5	127 (44.6)
	6	73 (25.6)
	7	10 (3.5)
Total (in school)		242 (84.9)
Not in school		43 (15.1)
**Total**		285 (100)

**TABLE 2:** Item pool of the Chilean, Brazilian and Ugandan version of the FESA.

			**Items Ugandan**
**Items**	**Subscale (Chilean sample)**	**Subscale (Brazilian sample)**	**sample**
I will find good work	work and education	work and education	x
I will acquire the things that I want	work and education	work and education	x
I will accomplish what I want to do with my life	work and education	work and education	x
I will find work that I enjoy	work and education	work and education	x
I will find stable work	work and education	work and education	x
I will always have enough to eat and live on	work and education	work and education	x
I will achieve the level of education that I want	work and education	work and education	x
I will feel satisfaction with myself	work and education	deleted	x
The money I earn with my spouse will be sufficient	work and education	deleted	x
My work will give me opportunities to feel proud of myself	work and education	deleted	x
I will regularly go to Mass or other religious services	church and community	church	x
I will participate in many church activities	church and community	church	
I will instill faith in my children, or nieces and nephews	not included	church	
I will do voluntary work in my community	not included	deleted after PCA	
I will be a leader in my community	church and community	deleted after PCA	x
I will get married	marriage and family	marriage	x
I will have children	marriage and family	children and family	x
I will get married before I am 25 years old	marriage and family	marriage	
My marriage will last forever	marriage and family	marriage	x
I will dedicate time to spend with my family	not included	children and family	x
My children will live in a time of peace	children’s future	children and family	x
My children will have a long life	children’s future	children and family	x
I will provide my children with a safe place to live	children’s future	children and family	x
I will have good health	health	health	x
I will have a healthy diet	health	health	x
I will participate in sports or another type of exercise	health	health	
I will have a long life	health	health	x

Of the 285 students, 91 (31.9%) had lost at least one parent, while 22 (7.7%) were complete orphans. Family income was low throughout the sample: 272 students (93.8%) reported financial struggles within their family during the past year. In addition, 104 (37.5%) families experienced hunger because of the lack of food at least once or twice during the month preceding the interview. All participants named Christianity as their religion.

### Results of the CFA

Confirmatory factor analysis with the remaining 21 items resulted in a model with an unacceptable model fit [Model fit: χ^2^(179, *n* = 231) = 613.033 (*p* < 0.001), TLI = 0.74, IFI = 0.79, CFI = 0.78, RMSEA = 0.11 (90%−CI = 0.10–0.12, PCLOSE = 0.00), SRMR = 0.06] including four items with low factor loadings between 0.188 and 0.487 (see Table 3). The factor structure of the original Chilean *FESA* (McWhirter and McWhirter, 2008) was thus deemed inappropriate for the Ugandan data.

**TABLE 3:** Factor loadings of the CFA (21 items, *n* = 231).

	**Work and**	**Church and**	**Marriage and**		**Children’s**
**Item (item number)**	**education**	**community**	**family**	**Health**	**future**
I will find work that I enjoy (5)	0.81				
I will acquire the things that I want (2)	0.60				
I will accomplish what I want to do with my life (1)	0.53				
I will find good work (3)	0.80				
I will find stable work (6)	0.78				
I will always have enough to eat and live on (7)	0.46				
I will feel satisfaction with myself (8)	0.52				
The money I earn with my spouse will be sufficient (9)	0.49				
My work will give me opportunities to feel proud of myself (10)	0.59				
I will achieve the level of education that I want (4)	0.74				
I will regularly go to Mass or other religious services (15)		0.34			
I will be a leader in my community (16)		0.19			
I will get married (11)			0.73		
My marriage will last forever (13)			0.80		
I will have children (12)			0.77		
I will have good health (17)				0.81	
I will have a healthy diet (19)				0.79	
I will have a long life (18)				0.56	
My children will live in a time of peace (21)					0.78
My children will have a long life (22)					0.73
I will provide my children with a safe place to live (20)					0.86

*Model fit: χ^2^ (179, *n* = 231) = 613.033 (*p* < 0.001), TLI = 0.74, IFI = 0.79, CFI = 0.78, RMSEA = 0.11 (90%−CI = 0.10–0.12, PCLOSE = 0.00).*

### Results of the PCA

The initial PCA with direct oblimin rotation of all 22 Ugandan *FESA* items revealed five components with eigenvalues over Kaiser’s criterion of 1. The KMO verified the sample adequacy of *N* = 279, with 0.88 for the whole scale (Hutcheson and Sofroniou, 1999). All KMO values for individual items were > 0.79, which is well above the acceptable limit of 0.50 (Field, 2011). Bartlett’s test of sphericity *X*^2^ (231) = 3340.828, *p* < 0.001, indicated that correlations between items were sufficiently large for PCA. Combined, the five extracted components were able to explain 66.34% of variance. Internal consistency for the complete 22-items version was α = 0.89. For a detailed presentation of the pattern matrix and the factor loadings of all 22 items after rotation refer to Table 4.

**TABLE 4:** Pattern matrix of PCA results for the Ugandan version of the FESA (all items, *n* = 279).

**Items (item number)**	**Pattern matrix**				
	**1**	**2**	**3**	**4**	**5**
I will acquire the things that I want (2)	0.777				
I will accomplish what I want to do with my life (1)	0.685				
I will feel satisfaction with myself (8)	0.668				
I will always have enough to eat and live on (7)	0.649				
I will have a healthy diet (19)	0.468				0.439
The money I will earn with my spouse will be sufficient (9)					
I will find work I enjoy (5)		0.817			
I will find stable work (6)		0.790			
I will achieve the level of education that I want (4)		0.777			
My work will give me opportunities to feel proud of myself (10)		0.680			
I will be a leader in my community (16)		0.677			
I will find good work (3)		0.642			
I will get married (11)			0.837		
I will have children (12)			0.818		
My marriage will last forever (13)			0.759		
I will dedicate time to spend with my family (14)			0.653		
I will provide my children with a safe place to live (20)			0.613		0.424
My children will live in a time of peace (21)			0.598		0.503
My children will have a long life (22)			0.515		
I will have a long life (18)				0.861	
I will have good health (17)				0.610	
I will regularly go to mass and other religious services (15)					0.735

The results of the PCA with all 22 items were discussed among a panel of experts with extensive experience in developmental and clinical research in Northern Uganda and led to the exclusion of three items: Item 9 (“The money I will earn with my spouse will be sufficient”) was deleted because it did not load on any factor. Many families still live off their own field crops or the exchange of self-grown crops and manual labor for other daily necessities within their communities. Item 15 (“I will regularly go to mass and other religious services”) was deleted as it was the only item dealing solely with religion. Also, most rural communities do not center their religious life around organized mass or religious services, as churches and religious meetings are hard to reach for the rural population. Many families organize their religious life in private prayer and worship sessions that were not sufficiently covered by item 15. Finally, item 18 (“I will have a long life”) was deleted from further analysis. During data collection, many participants and Ugandan clinical staff and teachers had mentioned that the prospects of a long life were nothing they could assess themselves as it was “entirely up to God himself to decide” how long they would live.

After deletion of these items, a second PCA with direct oblimin rotation was run with the remaining 19 items (*N* = 279). Sample adequacy was given (KMO = 0.89), and all KMO values for individual items were > 0.83. Bartlett’s test of sphericity *X*^2^(171) = 2899.760, *p* < 0.001, indicated that correlations between items were sufficiently large for PCA. The PCA with 19 items revealed three main components with eigenvalues > 1, which explained 61.12% of variance. No item loaded on more than one component. For a detailed look at the pattern matrix after rotation, refer to Table 5.

**TABLE 5:** Pattern matrix of PCA results for the Ugandan version of the FESA (19 items, *n* = 279).

**Items (item number)**	**Pattern matrix**		
	**1**	**2**	**3**
My marriage will last forever (13)	0.796		
My children will live in a time of peace (21)	0.775		
I will provide my children with a safe place to live (20)	0.769		
I will have children (12)	0.756		
I will get married (11)	0.751		
I will dedicate time to spend with my family (14)	0.695		
My children will have a long life (22)	0.670		
I will find work I enjoy (5)		0.811	
I will achieve the level of education that I want (4)		0.806	
I will find stable work (6)		0.771	
My work will give me opportunities to feel proud of myself (10)		0.685	
I will find good work (3)		0.671	
I will be a leader in my community (16)		0.593	
I will acquire the things that I want (2)			0.797
I will accomplish what I want to do with my life (1)			0.744
I will have good health (17)			0.706
I will feel satisfaction with myself (8)			0.673
I will have a healthy diet (19)			0.668
I will always have enough to eat and live on (7)			0.616

Main component labels were derived by consensus between the authors and in accordance with the factor labels of the original *FESA* (Chilean sample, McWhirter and McWhirter, 2008). The first component (7 items, α = 0.89) consisted of items relating to family life and children and accounted for 36.10% of variance. It was thus labeled *family and children*. The second component (6 items, α = 0.85) included items relating to education and work prospects and was labeled *work and education* accounting for 17.81% of the explained variance. The third component (6 items, α = 0.86) was composed of items describing a more general sense of future optimism, it was thus labeled *general future optimism*. The last component accounted for 7.21% of the explained variance. As expected, all main components were intercorrelated. The correlations between the three components ranged from 0.23 to 0.58, whereas the correlations between the components and the *FESA* sum-score ranged from 0.64 to 0.83 (see Table 6). The Ugandan FESA sum-score was calculated by adding all main component subscale-scores. The 19-item-version resulted in a scale ranging from 0 to 76. The current studies sample had a mean score of *M* = 63.04 (*SD* = 10.99, range = 21–76). The internal consistency of the whole scale was α = 0.89, the same as for the longer (*n* = 22 items) version of the first PCA. The distribution of the item values revealed that means were relatively high (between 2.55 and 3.60), covering the whole range of the scale from 0 to 4. For a detailed presentation of the item and subscale value distribution, please refer to Table 7.

**TABLE 6:** Main component correlations (Spearman’s rho, one-tailed) matrix for the Ugandan version of the FESA (*n* = 279).

			**General**	***FESA***
	**Family and**	**Work and**	**future**	**sum**
	**children**	**education**	**optimism**	**score^1^**
family and children	1			
work and education	0.229^∗∗^	1		
General future optimism	0.575^∗∗^	0.473^∗∗^	1	
*FESA* sum-score^1^	0.643^∗∗^	0.827^∗∗^	0.763^∗∗^	1

*^1^sum score of the Ugandan version of the *FESA*, 19 items. ^∗∗^*p* < 0.01.*

**TABLE 7:** Distribution (means, standard deviation and range) of the item, subscale, and total sum score values of the final PCA solution for the Ugandan FESA.

			**Standard-**
**Items (item number)**	**Range**	**Mean**	**deviation**
I will accomplish what I want to do with my life (1)	0–4	3.59	0.77
I will acquire the things I want (2)	0–4	3.30	0.96
I will find good work (3)	0–4	3.10	1.11
I will achieve the level of education I want (4)	0–4	2.87	1.35
I will find work I enjoy (5)	0–4	2.98	1.24
I will find stable work (6)	0–4	2.84	1.28
I will have always enough to eat and live on (7)	0–4	3.53	0.82
I will feel satisfaction with myself (8)	0–4	3.35	0.95
My work will give me opportunities to feel proud of myself (10)	0–4	3.22	1.12
I will get married (11)	0–4	3.47	0.99
I will have children (12)	0–4	3.56	0.87
My marriage will last forever (13)	0–4	3.40	0.99
I will dedicate time to spend with my family (14)	0–4	3.61	0.76
I will be a leader in my community (16)	0–4	2.55	10.51
I will have good health (17)	0–4	3.49	0.81
I will have a healthy diet (19)	0–4	3.54	0.75
I will provide my children with a safe place to live (20)	0–4	3.60	0.80
My children will live in a time of peace (21)	0–4	3.54	0.85
My children will have a long life (22)	0–4	3.50	0.85
**Scales**			
family and children (21, 20, 12, 11, 14, 22)	0–28	24.68	4.72
work and education (5, 4, 6, 10, 3, 16)	0–24	17.56	5.83
general future optimism (2, 1, 17, 8, 19, 7)	5–24	20.80	3.78
sum score Ugandan FESA	21–76	63.04	10.99

## Discussion

The present study used the combined item pool of the Chilean and the Brazilian *FESA* to administer the *FESA* to *N* = 279 Northern Ugandan adolescents. Based on their answers and a focus group discussion with local counselors and rural and urban teachers we developed a Ugandan version of the instrument. As expected, CFA revealed that the original five-factor structure of the Chilean *FESA* (McWhirter and McWhirter, 2008) was not a good fit for the Ugandan sample. In addition to the bad model fit, four items had very low factor loadings: The items “I will regularly go to mass and other religious services” and “I will be a leader in my community,” which load on the *church and community* factor in the Chilean original *FESA*, as well as the items “I will always have enough to eat and live on” and “The money I will earn with my spouse will be sufficient,” which load on the *work and education* factor in the Chilean original *FESA*. These low factor loadings are in accordance with the focus group feedback on the fit of these items in the Ugandan context and will be discussed further below.

Principal component analysis revealed a factor structure differing from the original Chilean and the Brazilian *FESA*. This result is in line with the observations of both prior *FESA* author teams, who argue that the contextual specificities of the participants’ background influence the measures’ factorial structure (McWhirter and McWhirter, 2008; Dutra-Thomé et al., 2015).

The final factorial structure of the Ugandan *FESA* includes the three factors *family and children*, *work and education*, and *general future optimism* and consists of 19 items. The scale had good internal consistency. The intercorrelations between the different subscales made theoretical sense, as both the *family and children* and *work and education* subscales correlated higher with the *general future optimism* scale than with each other.

The main difference between the Ugandan *FESA* and the original version of the questionnaire is the much simpler factorial structure of the measure. This was partly due to the deletion of items, which was based on statistical reasons and the focus group’s input. Many items that were deleted either before or after the PCA related to organized religious activities (“I will participate in many church activities”; “I will regularly go to mass and other religious services”) and the self-perceived choice of living a life influenced by religious faith (“I will instill faith in my children, nieces or nephews”). The participants in the focus groups argued that religious life in rural Ugandan communities is not as organized as the items imply. The focus group also stated that young adults are not presented with a choice of whether they want to lead a religiously influenced life or not, as the Christian faith is seen as a necessity in order to be accepted in most rural communities. The strong religious fatalism of the sample also led to the omission of the item “I will lead a long life,” as participants were not willing to assess “God’s choice” on how long they would live.

An additional three items were deleted on the grounds of the results of the focus group discussion, as they were not deemed appropriate for the rural Ugandan setting. The first item “I will participate in sports or another type of exercise” is not useful in a sample that mainly consists of families who work as agricultural fieldworkers. Most of these rural farmer families have neither the need nor the time to participate in leisurely physical exercise. The second item omitted was “I will do voluntary work in my community,” as communal work such as communal digging (a process in which neighbors take turns to help each other with fieldwork) is not considered voluntary by the community. Although the concept of community as an extended family has been in the process of changing since the civil war, helping community members is so deeply rooted in tradition that it is not seen as a choice. The last item from the overall item pool that was not elected for the Ugandan *FESA* was “I will get married before I’m 25 years old.” The focus group agreed that the item would be inadequate since the average age for marriage is very low in rural communities. Finally, after the PCA, the item “The money I will earn with my spouse will be sufficient” was deleted because it did not load on any factor. This might be due to the fact that the concept of sufficient wealth in rural northern Uganda is not directly tied to monetary salaries.

These changes in the overall item pool led to the three-factor solution, which explains sufficient variance. The main difference between the Ugandan *FESA* and the Original *FESA* as well as the Brazilian adaptation of the scale is the simpler factor structure with less distinct domains of future expectations. The domain dealing solely with *religion and community* as a separate entity of future expectations did not prove to be relevant for the Ugandan adolescents. As described above, leading a life that adheres to religious and community-based values is traditionally expected of adolescents and young adults in rural Ugandan communities. It thus seems out of question for adolescents to rate the importance of these traditional values for their personal future expectations.

Similarly, the concepts of *marriage and family* and *children’s future* which constitute separate factors in the original *FESA* seem to be much more intertwined in the Ugandan sample, as they make up the combined factor of *children and family in* the present study. One possible explanation is the above-mentioned fatalism of the sample’s participants: All of the *children’s future* items concern the personal assessment of how likely the adolescents deem the main prerequisites for a happy and safe life of their future offspring (such as peace) to be fulfilled.

The factor *work and education* presented a similar structure in the Ugandan sample compared to the original study. The only differences were the items (“I will acquire the things that I want”; “I will accomplish what I want to do with my life”; “I will feel satisfaction with myself”) which loaded instead onto the *general future optimism* factor. Interestingly, the items that loaded onto the *work and education* factor in the original *FESA* are the ones that are less concretely worded as specific career goals and are rather results of having a successful life in which one has obtained monetary and emotional stability. It seems as if the adolescents of the Ugandan sample had a narrower definition of career and education-related topics when discussing their future expectations. This may be due to the fact that some of these aspects of the future are attainable for adolescents growing up in rural Uganda without succeeding in a classic career based on further education. For example, if the agricultural work provides their future family with enough to feed themselves and exchange some field fruits for other needed goods, some of these goals are met without extensive education or a formal career.

This possible explanation in addition to the potential influence of fatalism on a more generic evaluation of the future in the present study’s sample explains the factor *general future optimism*, which includes the items mentioned above as well as the more generic health and nutrition-related items (“I will have good health”; “I will have a healthy diet”; “I will always have enough to eat and live on”). These items seem to describe more of a generally positive attitude toward the future in the Ugandan sample than to describe specific aspects of attained career or health goals as it was the case in the original *FESA* study. The overall feeling of an unforeseeable future, which is closely connected to fatalism (Peou and Zinn, 2015), might lead to a less differentiated view on future expectations. The factor *general future optimism* might thus be a good overall estimator of adolescents’ outlook onto their future.

One other sample characteristic worth noting is the fact that the overall future expectations of the adolescents were rather optimistic. Given the harsh living circumstances the participants grew up in Saile et al. (2013a,b, 2015) and the number of current stressors, such as lack of secure access to education or meals, this might surprise at first glance. However, a study by Nurmi (1988) was able to show that expecting the outbreak of nuclear war did not necessarily decrease the future expectations of Finnish adolescents. Adolescents who actually experienced a heightened threat of war showed more interest in their personal future than their less worried peers. Ugandan adolescents currently experience the influx of weapons and refugees from the conflicts of the neighboring countries South Sudan and the Democratic Republic of Congo. This could possibly influence their approach to evaluate and plan their own future. Additionally, it is important to note that stories of the past conflict are passed from the parents’ and grandparents’ generation to their offspring, emphasizing how much better the chances of a peaceful and long life are for the current generation of adolescents. It might well be that these tales of a better future influence the positive outlook on the future in the current sample. As the reconstruction of society after violent conflict is ideally linked to an increase in economic wealth for its people (Bozzoli et al., 2010a) this might also influence the positive future expectations.

On the single item level, it is noticeable that the items of the subscale *work and education* have the lowest means and largest standard deviations. It seems as if students’ future expectations vary mainly with regard to their outlook on career and education opportunities at this point in their lives. This makes sense, as the next 2 years will decide whether their families will allow them to attend secondary school, which will open up career opportunities beyond rural farming life. It would be interesting to follow up on students’ educational paths over the next few years, to see whether missed access to further education will impact their scores on the work and education subscale negatively. In contrast two items of the *family and community subscale* “I will dedicate time to spend with my family” and “I will provide my children with a safe place to live” have the largest means and vary the least over the sample. This is in accordance with the high importance of family life in Northern Uganda and the cultural expectation to “always provide for one’s family, no matter the circumstances” (focus group with rural teachers). Adolescents seem to be very sure that the family-related aspects of their future will be taken care of.

Overall, we were able to construct and evaluate a Ugandan version of the *FESA*. The item pool of the original *FESA* was mainly applicable to this new setting and seems to work in the changed cultural and socioeconomic setting of a post-conflict society in East-Africa. Although the resulting factor structure of the Ugandan *FESA* was much simpler and less detailed, it emphasized the main areas of adolescents’ future expectations, as described in the literature (e.g., Nurmi, 1991), of education and career as well as family life. The lack of more distinct domains within the adolescent’s future expectations might be due to the fact that future expectations are not habitually discussed or explored with teenagers and young adults in rural Uganda, as the focus group confirmed. According to Nurmi (1991) on influences on future expectations, parents and teachers are the most important role models for adolescents to acquire basic skills to develop future expectations. The teachers of the focus group agreed on the fact that most families’ daily life in rural Ugandan communities is controlled by the daily struggle to make ends meet in order to enable their children to attend school, be sufficiently fed, and safe. This struggle for daily survival leaves little to no capacity to focus on or develop any long-term plans for their offspring. This is in accordance with the conclusion of a review by Nurmi (1991) that adolescents from a lower socioeconomic background seem to have less detailed future plans than their wealthier peers. Jones and Brown (2005) argue that living under harsh social conditions with diminished contingencies between present behavior and future outcomes forces people to develop the ability to repeatedly and consistently manage the immediate situation to achieve a desirable future. This cognitive temporal bias toward a present orientation has also been linked to poverty and difficult access to formal education (Zimbardo and Boyd, 1999), which is the case for the Ugandan adolescents in this sample.

One other possible explanation is the fact that in times of societal and economic change such as those currently underway in Uganda, concrete future expectations might not only be harder to form but are also less adaptable than a broader approach to one’s future. Leccardi (2005) was able to show in a sample of Italian youth that in times of economic change, having broad guidelines instead of concrete future plans was the most feasible option to plan the future. Correspondingly, Vorhölter (2014) describes the difficulties of Northern Ugandan youth growing up with the ambivalence of a re-traditionalization discourse that idealizes the past and the growing influence and lure of the modernized world in the climate of the post-war years. This ambivalence might make it harder for adolescents to form very specific future expectations but support a broader, optimistic view. The more general factor structure of the Ugandan *FESA* might thus reflect the Ugandan adolescents’ ability to adapt flexibly to the quickly changing surroundings and newly developing social structure they grow up in.

The strengths of the present study lie in the large sample size of adolescents from a hard-to-reach rural post-conflict society as well as in the detailed input of three local focus groups. The evaluation sample is a good representation of adolescents living in the qualms and changes of an emerging post-conflict society, as the Ugandan teenagers interviewed in the study are currently struggling with the typical uncertainties and stressors of the post-war years, including economic hardship, an interrupted education history as well as major changes in family structure and societal rules. Due to the intensive exchange with the three focus groups before, during, and after data collection, we were able to gain important knowledge about the influence of the cultural specificities and societal changes that mark the post-conflict years in Uganda.

However, certain limitations must be noted. The sample consisted of adolescents who all share a very low socioeconomic status. As socioeconomic status has been linked to the extent of how detailed adolescents formulate their future expectations (Nurmi, 1991), the current Ugandan *FESA* structure should also be tested in a more affluent sample. Owing to their financial struggles, nearly all of the interviewed adolescents were still in primary school. It might well be that their future expectations will change once they enter permanently into work life and start having their own families. Entering these domains of adulthood might confront them with unrealistic expectations and might dampen their overall very optimistic outlook on their future or might lead to a more detailed look into the different domains of future expectations. As the sample focused on adolescents living in rural communities it should also be tested whether the resulting factorial structure of the Ugandan *FESA* can be applied to adolescents growing up in urban settings.

This study was able to show again that the item pool of the original *FESA* from the Chilean sample (McWhirter and McWhirter, 2008) is applicable, to a large extent, in a post-conflict society. After several modifications, the resulting Ugandan *FESA* provides an instrument with acceptable psychometric qualities to measure future expectations in Ugandan youth who have experienced civil war and a changing post-conflict society. The Ugandan FESA is especially useful for future research on the interaction between post-war stressors, psychopathology and future expectations in an emerging society that faces numerous changes and challenges. It would also be interesting to compare adolescents future expectations and their delay gratification in an experimental setting, as delay gratification has been linked to impulse control, excessive consumerism and risk behavior in young adults (Roberts and Martinez, 1997; Wulfert et al., 2002). This is especially relevant in Uganda with its very young population, growing middle-class and changing economic market. The results also suggest that an adaptation of the factor structure of the instrument is necessary when applying the *FESA* items in a new setting, as previously argued by Dutra-Thomé et al. (2015). Further studies should seek to replicate the Ugandan *FESA*’s factor structure in other post-conflict societies to assess whether there are cultural influences beyond the post-war setting alone. Future studies might also consider including other regions of Uganda or focus on specific subgroups defined by age, gender, or socioeconomic status to compare their mean sum-score as well as the factorial structure. A longitudinal study accompanying adolescents during the different stages of reaching adulthood could also give further insights into the stability of their future expectations.

## Ethics Statement

The ethics committee of the German Research Foundation (DFG), the ethics committee of Gulu University in Uganda (GUREC/05/12/15), and the National Council for Science and Technology in Uganda (UNCST- Ref: SS2329) approved of the study protocol. All participants and their primary caregivers were invited to an information meeting where the study’s objectives and procedures were explained. Written consent for participation and publication of at least one primary guardian and the participating adolescent were collected. No monetary incentives were given, but all participants received a snack during the interview. After completion of the study, all children who had participated in the study in 2010 and their guardians were invited to take part in a community training that involved fostering improved parenting skills (female guardians) and dealing with domestic violence and suicidal ideation (adolescents), independent of their participation in the data collection at the second (2012) or third (2016) wave.

## Author Contributions

CC and FN obtained the funding for the research. The study built upon a longitudinal survey on the developmental aspects of violence and mental health in this population designed by CC. LS was the main contributor of the specific design of this study, including data collection and analysis. LS, KG, and FN contributed to the data interpretation and the structure of the manuscript. LS drafted the manuscript. All authors, read and approved the final version of the manuscript.

## Conflict of Interest Statement

The authors declare that the research was conducted in the absence of any commercial or financial relationships that could be construed as a potential conflict of interest.
